# Differential expression of ST6GALNAC1 and ST6GALNAC2 and their clinical relevance to colorectal cancer progression

**DOI:** 10.1371/journal.pone.0311212

**Published:** 2024-09-30

**Authors:** Mohammed Saqif Ahmad, Maria Braoudaki, Shoib Sarwar Siddiqui

**Affiliations:** School of Life and Medical Sciences, University of Hertfordshire, Hatfield, United Kingdom; PLOS: Public Library of Science, UNITED KINGDOM OF GREAT BRITAIN AND NORTHERN IRELAND

## Abstract

Colorectal cancer (CRC) has become a significant global health concern and ranks among the leading causes of morbidity and mortality worldwide. Due to its malignant nature, current immunotherapeutic treatments are used to tackle this issue. However, not all patients respond positively to treatment, thereby limiting clinical effectiveness and requiring the identification of novel therapeutic targets to optimise current strategies. The putative ligand of Siglec-15, Sialyl-Tn (STn), is associated with tumour progression and is synthesised by the sialyltransferases ST6GALNAC1 and ST6GALNAC2. However, the deregulation of both sialyltransferases within the literature remain limited, and the involvement of microRNAs (miRNAs) in STn production require further elucidation. Here, we identified miRNAs involved in the regulation of *ST6GALNAC1* via a computational approach and further analysis of miRNA binding sites were determined. *In silico* tools predicted miR-21, miR-30e and miR-26b to regulate the *ST6GALNAC1* gene, all of which had shown significant upregulated expression in the tumour cohort. Moreover, each miRNA displayed a high binding affinity towards the seed region of *ST6GALNAC1*. Additionally, enrichment analysis outlined pathways associated with several cancer hallmarks, including epithelial to mesenchymal transition (EMT) and MYC targets associated with tumour progression. Furthermore, our *in silico* findings demonstrated that the *ST6GALNAC1* expression profile was significantly downregulated in CRC tumours, and its low expression correlated with poor survival outcomes when compared with patient survival data. In comparison to its counterpart, there were no significant differences in the expression of *ST6GALNAC2* between normal and malignant tissues, which was further evidenced in our immunohistochemistry analysis. Immunohistochemistry staining highlighted significantly higher expression was more prevalent in normal human tissues with regard to *ST6GALNAC1*. In conclusion, the integrated *in silico* analysis highlighted that STn production is not reliant on deregulated sialyltransferase expression in CRC, and *ST6GALNAC1* expression is regulated by several oncomirs. We proposed the involvement of other sialyltransferases in the production of the STn antigen and CRC progression via the Siglec-15/Sia axis.

## Introduction

Colorectal cancer (CRC) is one of the most prevalent and lethal malignancies worldwide, with patient diagnoses increasing each consecutive year [1]. Due to its malignant nature, CRC treatment is heavily reliant on early screening and detection to improve patient survival [2]. Current immune checkpoint blockade therapies have shown positive patient responses against programmed cell death protein-1 (PD-1), with blocking antibodies such as pembrolizumab and nivolumab being food and drug administration (FDA) approved for treatment in metastatic CRC patients with significant microsatellite instability (MSI) mutations [3]. However, not all patients respond positively to immunotherapeutic treatments, limiting clinical effectiveness and resulting in a poor prognosis. There is an urgent need to further identify therapeutic targets to optimise current treatment strategies for CRC.

The sialic acid-binding immunoglobulin-type lectin (Siglec)/sialic acid (Sia) axis is an immunoregulatory pathway that establishes immune tolerance to self-cells. More recently, this has been implicated in several cancer types, with Siglecs being focused upon as emerging therapeutic cancer targets [4]. Siglec-15 is one such promising immune checkpoint protein that exhibits distinct expression on cancer subpopulations compared to other immune checkpoints, such as programmed death-ligand 1 (PD-L1) [5]. The interaction of sialoglycan ligands upon Siglec-15 engagement prevents effective activation of T-lymphocytes [6]. The implications of Siglec-15 binding to sialoglycan ligands are those of inducing hypersialylation and promoting immunosuppression, consequently resulting in the immune evasion of tumour cells [7]. Several treatment strategies are being employed in the disruption of the Siglec-15/Sia axis. Current therapeutic strategies involve blocking antibodies (NC318) that are in clinical trials [8]. Similarly, another study highlighted the development of a monoclonal antibody (1-15D1) that had a high binding affinity to the Siglec-15 protein and was capable of stimulating T-cell response *in vitro* [9]. Moreover, other approaches for Siglec-15 disruption have utilised protein aptamers to assist in checkpoint blockade and have high affinity against the Siglec-15 protein [10]. Further to this, our previous study also highlighted the development of a small molecule inhibitor binding to the V-set binding domain to prevent sialoglycan binding [11]. Interestingly, upregulation of these Sia glycans has also been correlated with cancer-specific glycosylation and serves as a unique driver in cancer onset and progression [12]. More specifically, Siglec-15/Sia binding is dependent on Sialyl-Tn (STn), likely synthesised by glycosyltransferases such as *ST6GALNAC1*, which have shown aberrant expression in cancer [13]. *ST6GALNAC1*’s involvement in STn antigen presentation is frequently upregulated in multiple cancer types and has shown poor prognosis in CRC and prostate cancer (PCa) [14,15]. Similarly, *ST6GALNAC2* participates in STn antigen generation to a lesser extent and demonstrates high expression in CRC, and exhibits advanced cancer progression in follicular thyroid carcinomas (FTC) [16,17]. Targeting sialyltransferases such as *ST6GALNAC1* and *ST6GALNAC2* may provide anti-tumour immunity and a greater response in current immune checkpoint blockade therapies. However, evidence regarding miRNAs involved in their regulation and STn production in the literature remain limited.

MiRNAs are small single-stranded RNA molecules capable of modulating gene expression via 3’ untranslated region (UTR) binding post-transcriptionally [18]. Furthermore, the interplay of miRNAs enhances cancer hallmarks and its regulation, and correlates with targeting key genes involved in promoting angiogenesis, cancer proliferation, and metastasis [19,20]. Similarly, the Siglec/Sia axis can also be modulated via miRNA expression. The upregulation of miR-135b and miR-182 directly targets *ST6GALNAC2* via the phosphoinositide 3-kinase/protein kinase B (PI3K)/AKT signalling pathway, enhancing chemoresistance in CRC [21]. The crosstalk between miRNA expression patterns and the Siglec-15/Sia axis could underline certain dysregulated mechanisms.

In the current study, a multiomics approach was adopted to investigate the roles of *ST6GALNAC1* and *ST6GALNAC2* in mediating the Siglec/Sia axis and its clinical relevance to CRC tumorigenesis at the gene and protein levels. We further highlighted enriched pathways associated with cancer hallmarks and identified possible signalling pathways related to tumour onset. Moreover, we addressed the impact of each corresponding gene on the infiltration of myeloid cells and their association with prominent immune checkpoints and immune function. This may provide insights into highlighting their roles in cancer progression.

## Methodology

### Search Tool for the Retrieval of Interacting Genes/Proteins (STRING)

The STRING database identifies known and predicted protein-protein interactions (PPI) and displays direct associations via computational data mining. The STRING 11.0 (https://version-11-0b.string-db.org/cgi/input?sessionId=brxKfZgAG9Au&input_page_show_search=on; accessed 2 August 2023) software highlighted PPI relationships in *ST6GALNAC1* and *ST6GALNAC2*. The development of the full PPI network included both functional and physical protein associations with ≤ 10 protein interactors. Additionally, each individual protein queried and ≤ 20 protein interactors were included in the second expansion of the framework with a high confidence interval, with ≥ 0.700 considered significant.

### GeneMANIA

The GeneMANIA database (https://genemania.org/; accessed 26 July 2023) predicts functional information based on corresponding genes and gene datasets. The gene framework highlights functional communication between genes via criterion comprising of physical interactions, co-expression, predicted datamining, co-localisation, genetic interactions, and common pathway interactions with respect to *ST6GALNAC1* and *ST6GALNAC2*. Correlating genes with a high degree of association were also represented [22].

### UALCAN

The UALCAN database (http://ualcan.path.uab.edu; accessed 26 July 2023) is used as a tool for cancer transcriptomics. Utilising TCGA genomic data for analysis, the mRNA expression of *ST6GALNAC1* (ENSG00000070526) and *ST6GALNAC2* (ENSG00000070731) were compared for normal and colon adenocarcinoma (COAD) cohorts. Similarly, gene expression of identified protein targets obtained from the STRING analysis were determined and compared between normal and COAD subgroups. Additionally, predicted miRNA candidates involved in gene regulation were also compared for normal and COAD cohorts. Statistical significance of gene expression data (transcript per million; TPM) generated by the UALCAN database for box plot construction was assessed via a Welch’s t-test PERL script encoded to identify significant differences between cohorts based on clinicopathological features [23]. P < 0.05 was considered statistically significant.

### *In silico* miRNA datamining

*In silico* analysis tools were utilised to determine common miRNA targets predicted to modulate *ST6GALNAC1* expression and possible clinical relevance to CRC across several databases. Venn diagrams (Venny 2.1.0) (https://bioinfogp.cnb.csic.es/tools/venny/; accessed 28 July 2023) were constructed using three separate miRNA prediction databases including TargetScan (https://www.targetscan.org/vert_80/; accessed 28 July 2023), MiRSystem (http://mirsystem.cgm.ntu.edu.tw/index.php; accessed 28 July 2023), and MiRWalk (http://mirwalk.umm.uni-heidelberg.de/; accessed 28 July 2023), and were further cross-referenced. Furthermore, common miRNA candidates were also determined for the identified *in silico* targets that were shown to interact with *ST6GALNAC1* and *ST6GALNAC2*. The most frequent miRNA hits associated with *ST6GALNAC1* were further explored for predicted binding sites via statistical folding of nucleic acids and studies of regulatory RNAs (Sfold) software (https://sfold.wadsworth.org/cgi-bin/starmir.pl; accessed 13 September 2023).

### Gene Set Enrichment Analysis (GSEA)

GSEA of *ST6GALNAC1* and *ST6GALNAC2* were obtained via the TCGA dataset (TCGA, PanCancer Atlas) from cBioPortal for Cancer Genomics (http://cbioportal.org/ accessed on 19 September 2023). CBioPortal is offered as an open access repository for interactive omics patient data. The dataset for COAD tumours was selected, comprising of a total of 524 patient samples. Each of the corresponding genes were submitted as the queried gene and modified to include the differential mRNA expression relative to the normal cohort with a z-score threshold of ± 2.0. Following this, the mRNA comparative data between the altered (differentially expressed group) and unaltered group (unchanged expression group) was separated. Following this, the altered group containing only the significant differentially expressed genes between the normal and COAD subgroups were selected, which also included the query genes *ST6GALNAC1* and *ST6GALNAC2*, respectively. The number of differentially expressed genes for each dataset were recorded as 6667 genes for the *ST6GALNAC1* dataset and 4331 genes for the *ST6GALNAC2* dataset and exported as TSV files. The datasetswere uploaded into the GSEA v4.3.2 software as rnk files (https://www.gsea-msigdb.org/ accessed on 19 January 2023) and enriched pathways and cancer hallmarks were identified (FDR < 0.25 and p < 0.05).

### TISIDB

The TISIDB database (http://cis.hku.hk/TISIDB/index.php; accessed on 26 July 2023) provides an integrated repository for identifying interactions between tumours and the immune system. The TISIDB database collates information via several datasets including TCGA transcriptomics and clinical data pertaining to multiple cancer types. The Spearman’s rank correlation between the abundance of macrophages, activated CD4^+^ T-cells, activated CD8^+^ T-cells, regulatory T-cells (Tregs), and monocytes were assessed in conjunction with the expression of both sialyltransferases, *ST6GALNAC1* and *ST6GALNAC2*, in COAD tumours [24].

### Tumour IMmune Estimation Resource (TIMER) analysis

The TIMER database (https://cistrome.shinyapps.io/timer/, accessed on 1 August 2023) serves as an extensive platform to systematically analyse immune infiltrates over several cancer types. For this study, specific parameters relating to the correlation of immune checkpoint protein genes, including *SIGLEC15*, *PDCD1*, *CD274*, cytotoxic T-lymphocyte associated protein 4 (*CTLA4*), T-cell immunoreceptor with Ig and ITIM domains *(TIGIT)*, and Lymphocyte-activation gene 3 (*LAG3*), were all compared with the abundance of *ST6GALNAC1* and *ST6GALNAC2*. Spearman’s rank correlation coefficient and p < 0.05 for log2 TPM was used to determine the statistical significance of COAD tumours [25].

### Kaplan-Meier Plotter (KM PLOTTER)

The Kaplan-Meier plotter (https://kmplot.com/analysis/, accessed on 26 July 2023) database tool was used to identify the prognostic association between the genomic expression data for *ST6GALNAC1* and *ST6GALNAC2*, along with corresponding patient survival outcomes in COAD tumours. The prognostic expression of each highlighted sialyltransferase in CRC patients was analysed over a period of time based on overall survival criterion (OS), relapse-free survival (RFS), post-progression survival (PPS), hazard ratio (HR), 95% confidence interval (CI), and Log-rank P-value [26].

### Immunohistochemistry (IHC)

Colorectal cancer tissue array (BC05023a) obtained from Biomax (TissueArray.Com LLC, Maryland, USA), containing 54 cores (18 COAD, 18 cancer adjacent colon tissue (AT), and 18 adjacent normal colon tissue (NAT), was subjected to histological staining. Deparaffinisation was achieved by treating the sections with histoclean/ethanol followed by antigen retrieval via sodium citrate treatment, and subsequently washed in 0.025% triton-x/PBS and incubated in 3% hydrogen peroxide:PBS for a period of 15 min. The slides were washed an additional three times with Triton-X100/PBS for 5 min. Blocking of non-specific binding sites was performed using 5% BSA/PBS, and the sections were then incubated in a humidity chamber for 1h at room temperature (RT) whilst wrapped in parafilm. The slides were incubated overnight at 4°C with the primary *ST6GALNAC1* antibody (1:50) (Proteintech Group, Inc., Manchester, UK) and *ST6GALNAC2* antibody (1:100) (Life Technologies Limited, Renfrewshire, UK) respectively. The slides were washed with 0.025% Triton X-100/PBS, followed by incubation with a biotin-labelled anti-rabbit secondary antibody. Subsequently, the sections were washed using a 0.025% Triton X-100/PBS wash step and 1h incubation with a streptavidin-HRP conjugate. Visualisation of both *ST6GALNAC1* and *ST6GALNAC2* antibody staining was achieved via addition of DAB solution (Zytomed Systems GmBH, Berlin, Germany) and haematoxylin as the nuclei stain for a period of 10 min at RT. Quantification of the staining was conducted via an AxioCam Hrc (Zeiss Microscopy, Oberkochen, Germany) microscope at x4 and x10 magnifications. Both normal and malignant tissues were compared via the unpaired student’s t-test.

## Results

### *ST6GALNAC1* and *ST6GALNAC2* gene and protein-protein molecular networks

**Fig 1:**
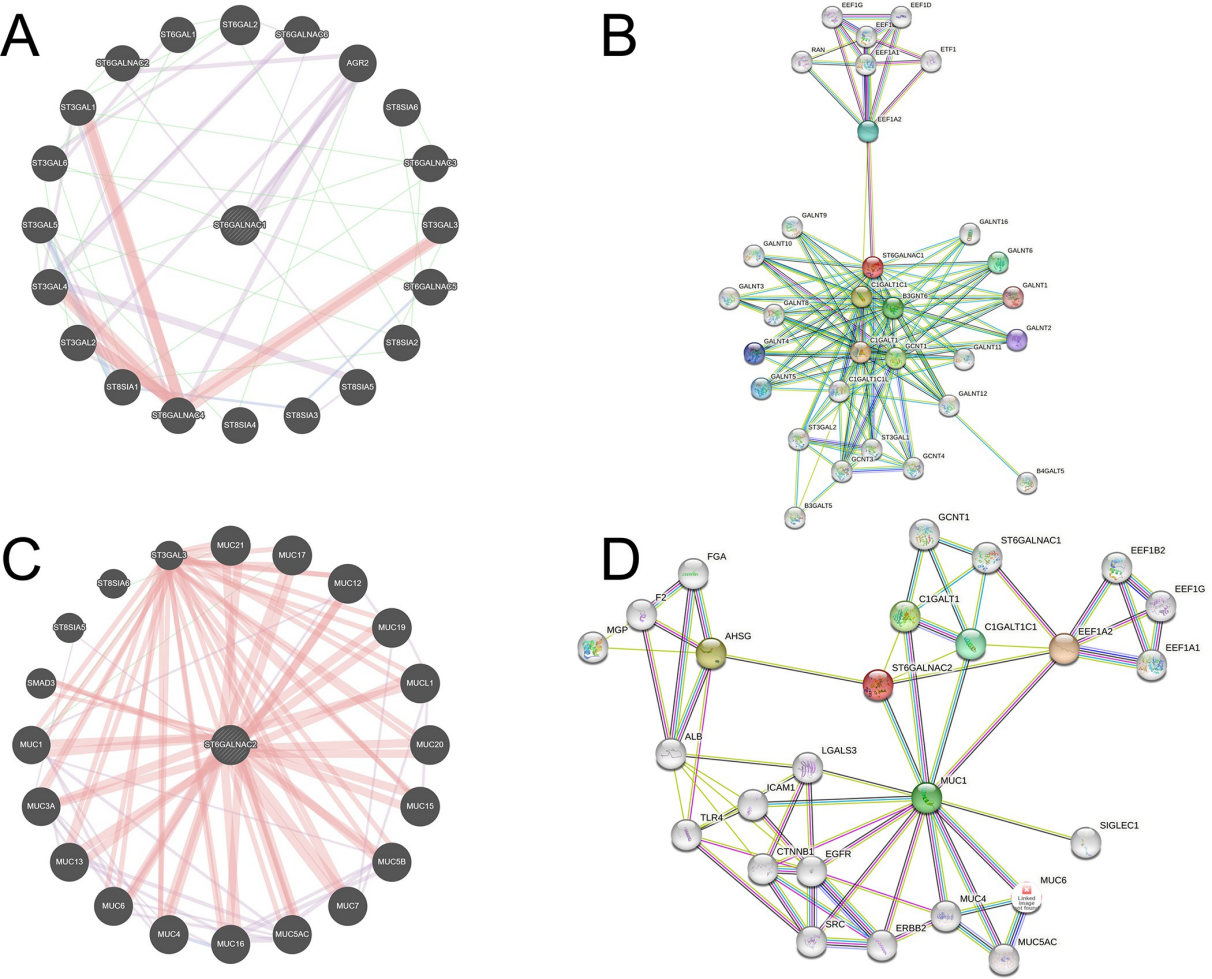
*ST6GALNAC1* and *ST6GALNAC2* share significant gene and protein-protein interactions and may highlight key novel targets. (**A**) S*T6GALNAC1* gene framework predicts functional information based on corresponding genes and gene datasets. (**B**) *ST6GALNAC1* STRING framework highlighting possible PPI from interacting proteins and direct associations via computational datamining. The framework is developed with a confidence interval of ≥ 0.700. (**C**) S*T6GALNAC2* gene framework predicts functional information based on corresponding genes and gene datasets. (**D**) *ST6GALNAC2* STRING framework highlighting possible PPI from interacting proteins and direct associations via computational datamining. The framework is developed with a confidence interval of ≥ 0.700.

The STRING and GeneMANIA databases highlight predicted genes and PPI between interacting queried proteins/genes. The *ST6GALNAC1* gene framework, produced by GeneMANIA, shows co-expression (purple) with anterior gradient 2 (*AGR2*) (Fig 1A) (Fig 1A). The *ST6GALNAC1* PPI network (Fig 1B) has shown multiple hits that correspond with N-acetylgalactosaminyltransferase (GALNT) enzymes and other sialyltransferases. Likewise, the average local clustering coefficient (ALCC) (0.83) shows a strong correlation with multiple protein targets, and the PPI enrichment score upon normalization shows significant node interactions at the highest confidence interval (< 1.0 x 10–16) (S1 Table). Predicted gene communication involving *ST6GALNAC2* has shown significant physical interactions with the MUC gene family (Fig 1C). The *ST6GALNAC2* PPI framework (Fig 1D) has also demonstrated a comprehensive predicted interaction clustering of several protein targets with 26 nodes. The ALCC score (0.708) and PPI enrichment (1.09 x 10^−5^) (S1 Table) show strong correlation between several protein targets and with primary interactions associated with α-2-HS-glycoprotein (AHSG). Furthermore, both sialyltransferases show interactions with a cluster of EEF1 enzymes, particularly with EEF1A2, and communication with Core-1 synthase-glycoprotein-*N*-acetylgalactosamine 3-b-galactosyltransferase-1 (C1GALT1) and C1GALT1 Specific Chaperone 1 (C1GALT1C1) (Fig 1B and 1D).

### Candidate miRNAs predicted in gene regulation and possible binding sites

**Fig 2:**
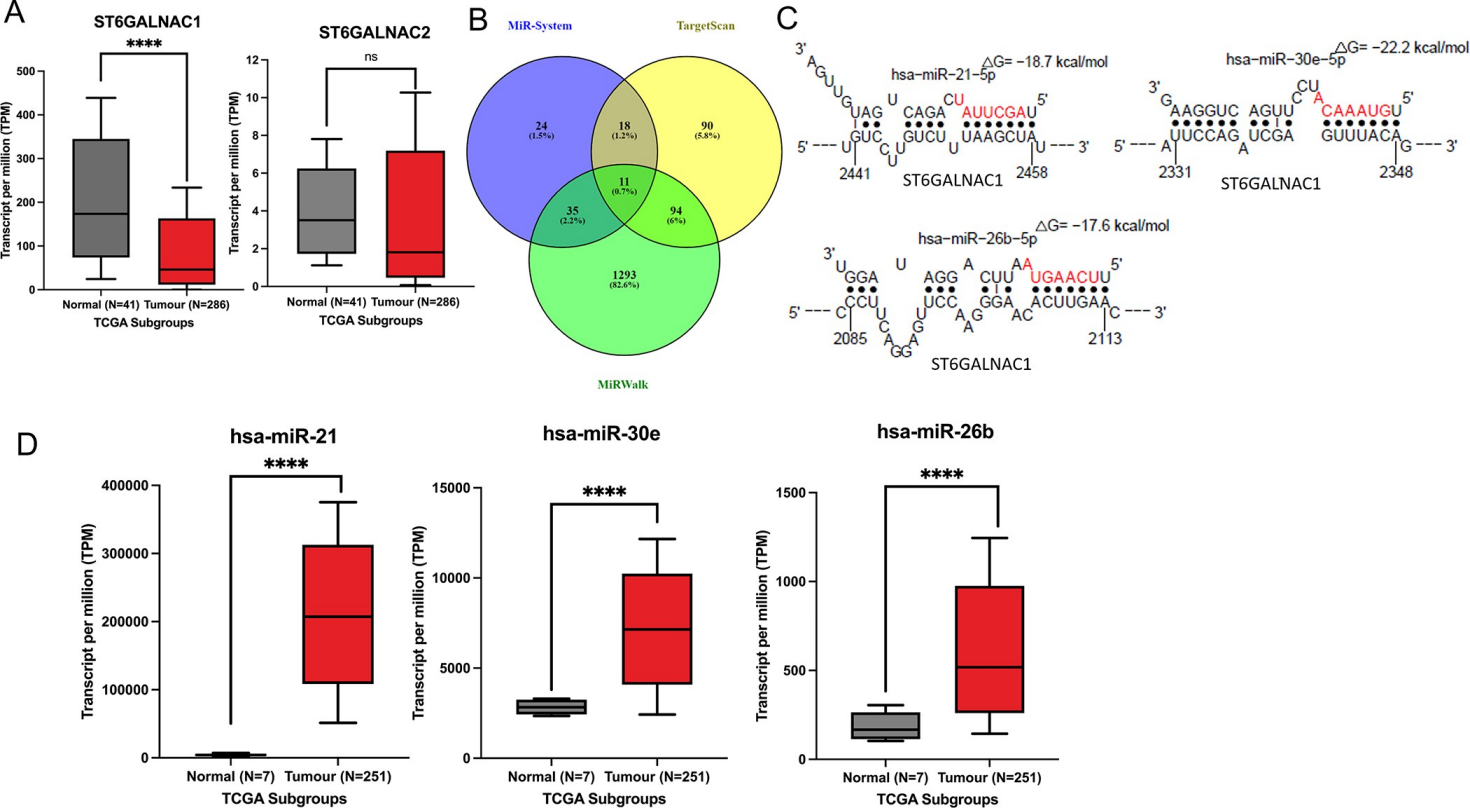
*In silico* analysis demonstrates that sialyltransferase expression is downregulated in COAD tumours. (**A**) TCGA genomic data demonstrating the mRNA expression of *ST6GALNAC1* for both normal and colon adenocarcinoma (COAD) cohorts. Welch’s t-test PERL script. **** P < 0.0001 (**B**) *In silico* analysis tools were utilised to construct Venn diagrams identifying common miRNA targets predicted to modulate *ST6GALNAC1* expression. (**C**) The most frequent miRNA hits associated with *ST6GALNAC1* were further explored for predicted binding sites via the Sfold software. (**D**) TCGA genomic data demonstrating the mRNA expression of candidate miRNAs with the highest binding affinities for normal and colon adenocarcinoma (COAD) cohorts. Welch’s t-test PERL script; **** P < 0.0001.

UALCAN TCGA datamining highlighted the sialyltransferase expression profile of *ST6GALNAC1* and *ST6ALNAC2* in CRC (Fig 2A). *ST6GALNAC1* shows significant gene downregulation in the tumour cohort of 46.056 TPM in comparison to the normal subgroup with a mean expression of 173.703 TPM (p < 0.0001). Additionally, UALCAN expression analysis shows no significant change in the expression of *ST6GALNAC2* between normal (3.509 TPM) and tumour (1.813 TPM) cohorts. Similarly, the gene expression of the predicted targets *AGR2* and *AHSG* were also determined between normal and CRC tumour groups. UALCAN expression identified significant expression of *AHSG* in the tumour subgroup (0.032 TPM) in comparison to the normal tissue subgroup (0.00 TPM) (S1B Fig). In contrast, *AGR2* had shown no significant difference in the expression of both subgroups. Furthermore, only *ST6GALNAC2* displays enhanced promoter methylation in the tumour cohort (S2B Fig). Moreover, the Pearson correlation between both *ST6GALNAC1* and *ST6GALNAC2* expression in CRC shows a low association between the two genes, but the relationship has been shown to be significant (S3 Fig).

To identify the miRNAs involved in regulating the gene expression of *ST6GALNAC1*, several databases were compared to identify the most frequent hits (Fig 2B). Of the multiple miRNAs cross-referenced, a total of 11 were predicted to be involved in the regulation of *ST6GALNAC1* based on targetscan, miRsystem and miRwalk databases (Fig 2B) (S2 Table). We have found that none of the 11 commonly deregulated miRNAs (Fig 2B) were regulating *ST6GALNAC1* as their expression levels seem to be downregulated as well. Hence, we focused on the miRNAs found common between Targetscan and miRsystem, and we found that miR-21-5p, miR-30e-5p and miR-26b-5p as potential regulators of *ST6GALNAC1* promoting CRC progression when overexpressed (Fig 2C and 2D). All three miRNAs were also shown to regulate ST6GALNAC1 by miRPathDB database as well (data not shown). Moreover, common miRNAs from the TargetScan database were identified between regulating *AHSG* and *AGR2* in association with *ST6GALNAC1* and *ST6GALNAC2* (S4A Fig). Although no singular miRNA was shown to regulate all four genes, common miRNAs across other predicted protein targets were also determined. The common miRNA element that correlated between the targets *GALNT3*, *GALNT8*, *B3GNT6* and *ST6GALNAC1* identified hsa-miR-30a-5p to be involved in gene regulation (S4B Fig). Similarly, predicted targets *C1GALT1*, *C1GALT1C1*, *AHSG* and *ST6GALNAC2* were compared for any common miRNA elements, although none were identified (S4C Fig). Further to this, predicted miRNAs involved in *ST6GALNAC1* expression were further investigated, and the binding affinities to key binding sites were identified (Fig 2C). MiRNA activity for gene regulation typically occurs at the 3’ end of the UTR. MiR-21-5p exhibited a high binding affinity (-18.700 kcal/mol) towards the seed region of the target *ST6GALNAC1* mRNA strand at position 2441–2458. Similarly, the predicted binding of miR-30e-5p and miR-26b-5p also displayed high binding affinities to the target mRNA strand at -22.200 kcal/mol and -17.600 kcal/mol, respectively. MiR-30e-5p had a predicted binding site at position 2331–2348. In contrast, miR-26b-5p had demonstrated a predicted binding site at position 2085–2113, all of which indicated a strong association between nucleotide bases.

To understand the role of these identified candidate miRNAs and their relevance in relation to CRC (Fig 2D), UALCAN data demonstrated significantly upregulated expression of all three miRNAs in the COAD tumour subgroup. MiR-21 displayed a mean expression of 207,192.191 TPM in comparison to normal colon tissues. This was also similarly observed by the mean expression of miR-30e with 7142.537 TPM and a mean expression of 518.048 TPM for miR-26b in comparison to the normal colon tissue cohort.

### GSEA analysis of sialyltransferases and cancer hallmarks

**Fig 3:**
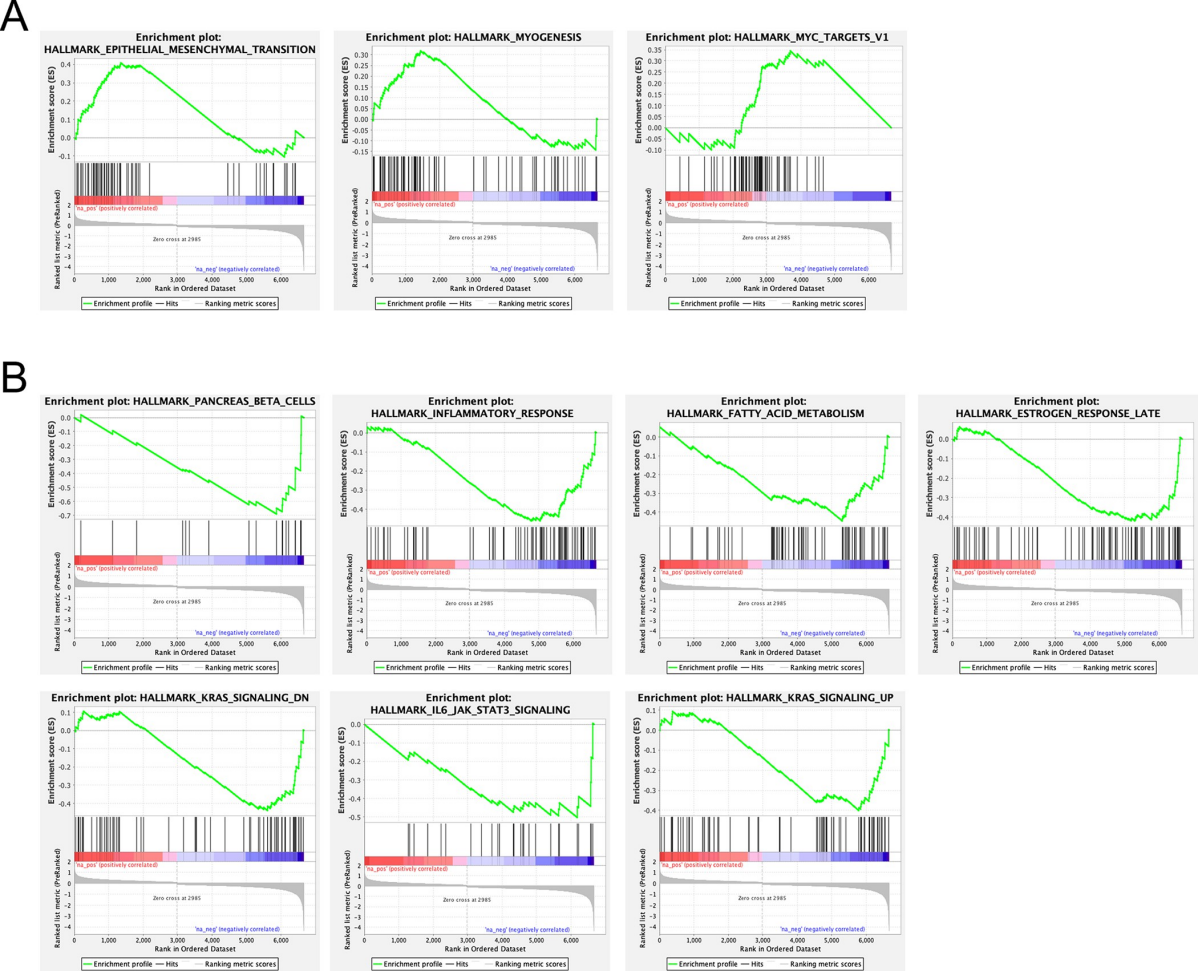
GSEA of *ST6GALNAC1* identified significant enrichment between DEG’s and the CRC phenotype. GSEA of *ST6GALNAC1* was obtained via the TCGA dataset (TCGA, PanCancer Atlas) from cBioPortal outlining DEG’s and a total of 47 gene sets associated with cancer hallmarks. (**A**) The enrichment of three gene sets was identified as being upregulated. FDR < 0.25 and p < 0.05. (**B**) Enrichment of seven gene sets was identified as being downregulated. FDR < 0.25 and p < 0.05.

**Fig 4:**
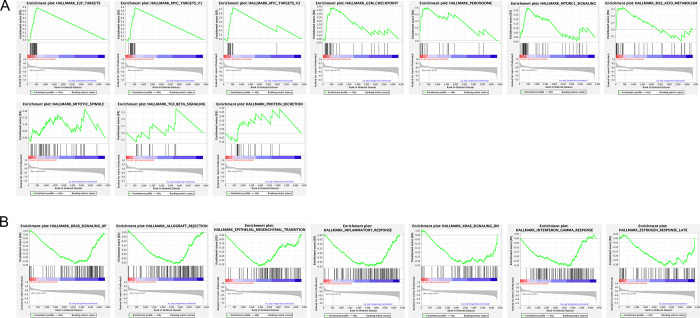
GSEA of *ST6GALNAC2* identified significant enrichment between DEG’s and the CRC phenotype. GSEA of *ST6GALNAC2* was obtained via the TCGA dataset (TCGA, PanCancer Atlas) from cBioPortal outlining DEG’s and a total of 42 gene sets associated with cancer hallmarks. (**A**) *ST6GALNAC2* highlighted 10 significantly enriched gene sets. FDR < 0.25 and p < 0.05. (**B**) Significant enrichment of seven gene sets was identified as being significantly downregulated. FDR < 0.25 and p < 0.05.

To identify various enriched pathways relating to *ST6GALNAC1* and *ST6GALNAC2* and cancer hallmarks, the GSEA of the TCGA, PanCancer Atlas dataset with a specific focus on significantly differentially expressed (DE) genes relating to CRC were highlighted with respect to *ST6GALNAC1* (Fig 3) and *ST6ALNAC2* (Fig 4). Out of a total of 47 gene sets displayed for *ST6GALNAC1* and the CRC phenotype, three gene sets were identified as significantly upregulated, whilst seven gene sets were identified as being significantly downregulated with respect to CRC (FDR < 0.25 and p < 0.05) (Fig 3A and 3B, S3 Table). Significant enrichment of EMT, MYC targets, and hallmarks related to myogenesis were identified as being significantly enriched. Additionally, enriched gene sets that had shown downregulation including the inflammatory response, IL-6 mediated JAK/STAT3 signalling, and deregulated KRAS signalling, among others. Similarly, *ST6GALNAC2* highlighted 10 significantly enriched gene sets as being upregulated (FDR < 0.25 and p < 0.05) (Fig 4A), and seven gene sets that were significantly enriched as being downregulated out of a total of 42 gene sets (S4 Table). Upregulated gene sets included the enrichment of E2F targets, MYC targets, the G2M checkpoint, mTOR signalling, and other pathways (Fig 4A). Downregulated gene sets that were also enriched included KRAS signalling, allograft rejection, EMT, inflammatory response, and IFNγ response, among other pathways (Fig 4B).

### Sialyltransferase expression correlates to the abundance of myeloid cells

**Fig 5:**
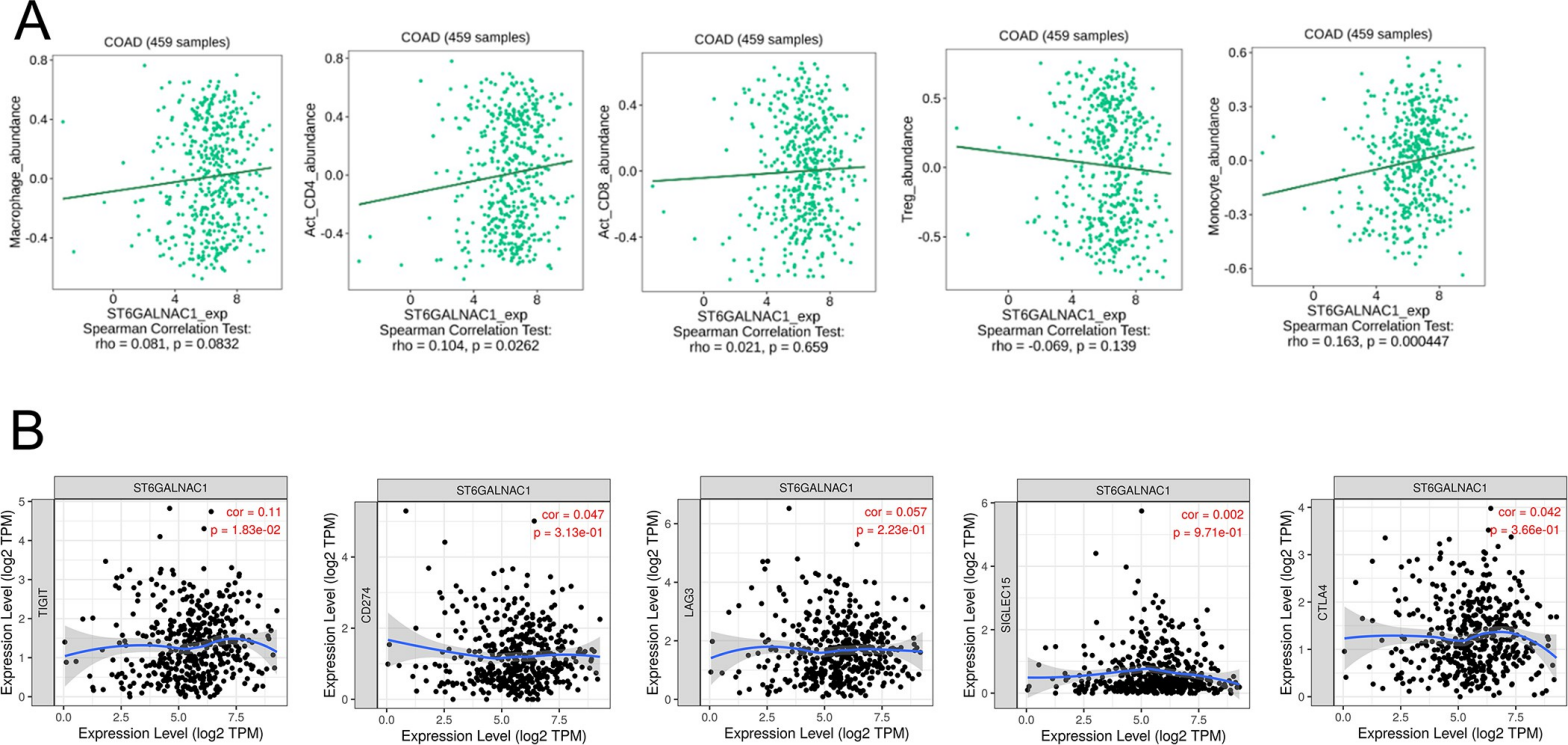
*ST6GALNAC1* correlates with CD4^+^ T-lymphocytes and monocyte populations but shows no significant correlation with other pro-tumorigenic immune checkpoints. (**A**) SRCC between the abundance of macrophages, activated CD4^+^ T-cells, activated CD8^+^ T-cells, regulatory T-cells (Tregs), and monocytes were assessed in conjunction with the expression of *ST6GALNAC1* in COAD tumours. P < 0.05 was considered statistically significant. (**B**) The association between the abundance of *ST6GALNAC1* and pro-tumorigenic immune checkpoints was determined via TIMER analysis.

**Fig 6:**
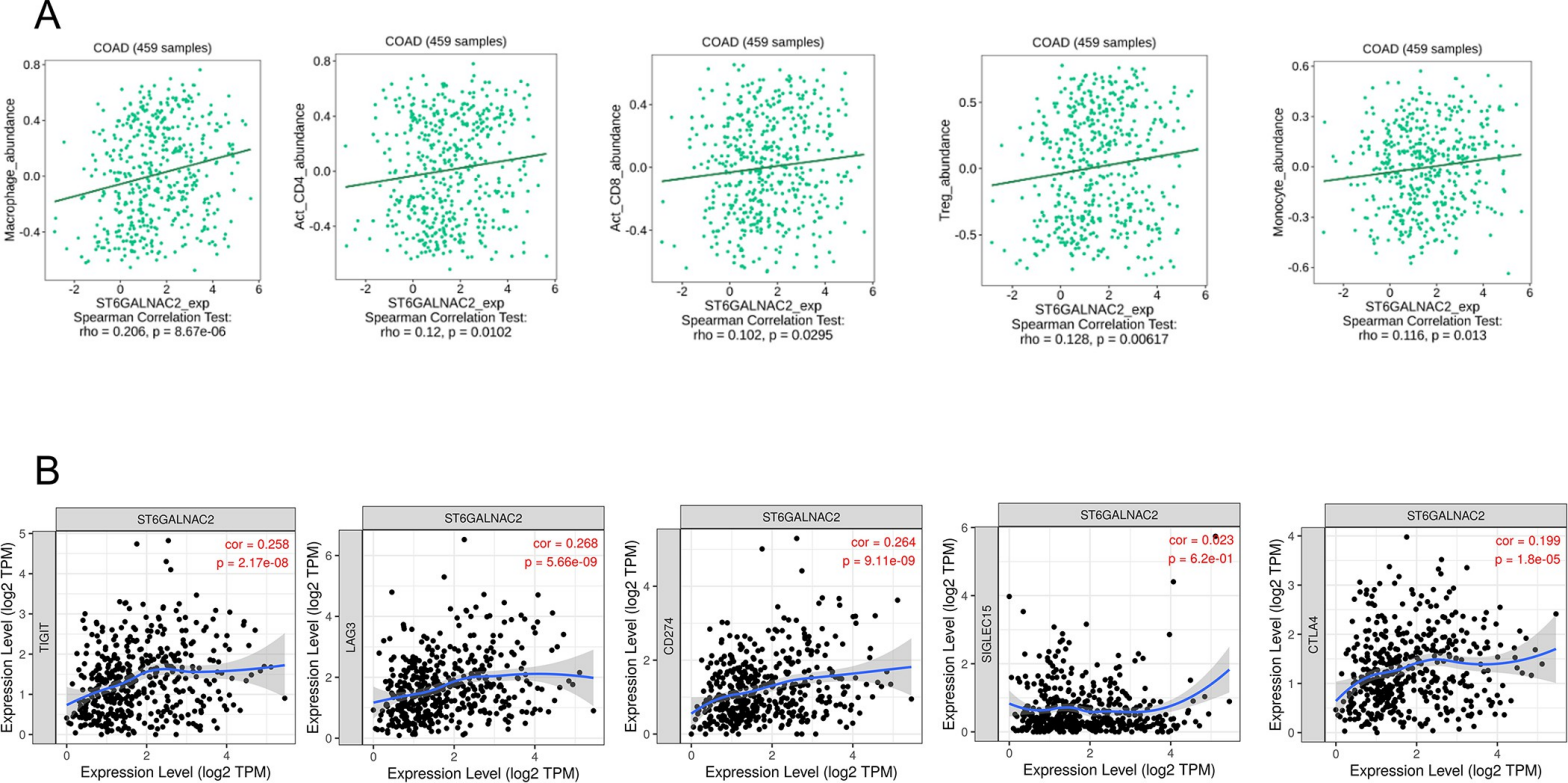
*ST6GALNAC2* correlated with the abundance of several infiltrating immune cell populations and displayed an association with most pro-tumorigenic immune checkpoints, excluding Siglec-15. (**A**) SRCC between the abundance of macrophages, activated CD4^+^ T-cells, activated CD8^+^ T-cells, regulatory T-cells (Tregs), and monocytes were assessed in conjunction with the expression of *ST6GALNAC2* in COAD tumours. P < 0.05 was considered statistically significant. (**B**) The association between the abundance of *ST6GALNAC2* and pro-tumorigenic immune checkpoints was determined via TIMER analysis.

To determine the role of both sialyltransferases and immune infiltration *in situ*, the abundance of immune infiltrating cell populations correlating with *ST6GALNAC1* and *ST6GALNAC2* expression in colon adenocarcinomas (COAD) was determined (Figs 5A and 6A). Spearman’s rank correlation coefficient (SRCC) significantly associated the abundance of activated CD4^+^ T-lymphocytes and monocyte populations with *ST6GALNAC1* expression (Fig 5A). In comparison, *ST6GALNAC2* expression correlated with the abundance of several infiltrating immune cell populations including activated CD4^+^ T-lymphocytes, activated CD8^+^ T-lymphocytes, Tregs, monocytes, and macrophages, with the most significant SRCC correlation with the latter (0.206) (Fig 6A). Additionally, the association between the abundance of both *ST6GALNAC1* and *ST6GALNAC2* and pro-tumorigenic immune checkpoints were also determined via TIMER analyses (Figs 5B and 6B). *ST6GALNAC1* expression showed no significant correlation with other pro-tumorigenic immune checkpoints (Fig 5B). However, *ST6GALNAC2* expression displayed an association with most pro-tumorigenic immune checkpoints, excluding Siglec-15 (Fig 6B).

### Dysregulated sialyltransferase expression correlated with poor clinical outcomes

**Fig 7:**
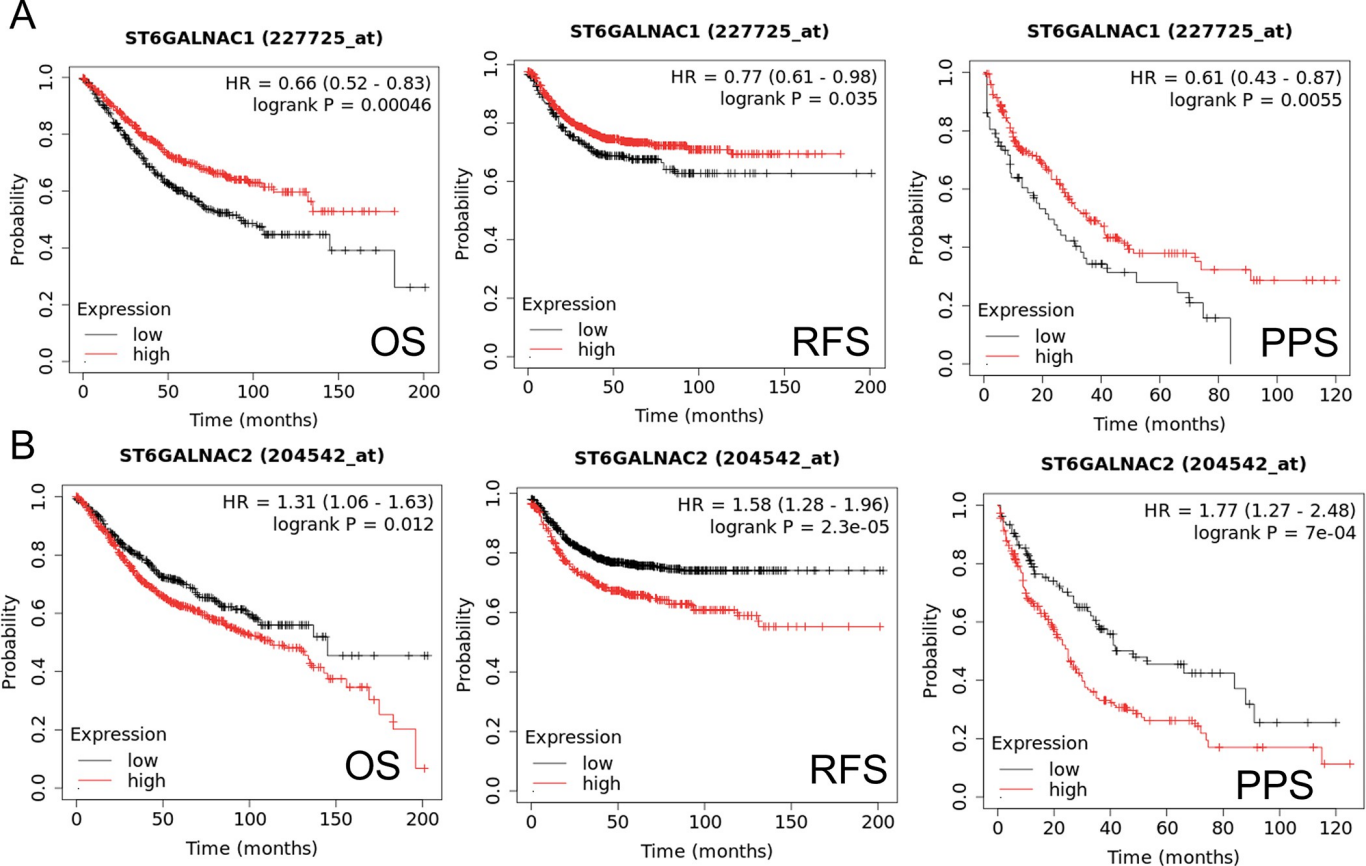
*ST6GALNAC1* shows low expression to be significantly correlated to poor prognosis based on survival criteria. High *ST6GALNAC2* expression was significantly associated with poor prognosis under all survival criteria. Only miR-940 OS criteria highlighted that low expression was associated with poor prognosis. (**A**) Survival curves outlining *ST6GALNAC1* expression with regard to patient survival data outlining overall survival (OS), post-progression survival (PPS), and relapse-free survival (RFS) criteria. P < 0.05 was considered statistically significant. (**B**) Survival curves outlining *ST6GALNAC2* expression with regard to patient survival data outlining overall survival (OS), post-progression survival (PPS), and relapse-free survival (RFS) criteria. P < 0.05 was considered statistically significant.

To correlate the expression of *ST6GALNAC1* and *ST6GALNAC2* to patient survival data, survival curves outlining overall survival (OS), post-progression survival (PPS), and relapse-free survival (RFS) were determined (Fig 7). *ST6GALNAC1* shows low expression that is significantly correlated to poor prognosis based on OS criteria (p < 0.05), the RFS criterion (p < 0.05), and PPS (p < 0.05) survival in CRC patients (Fig 7A). Moreover, high *ST6GALNAC2* expression was significantly associated with poor prognosis in CRC patients under all survival criteria (p < 0.05) (Fig 7B).

### ST6GALNAC1 protein expression is more prevalent in normal colon tissues

**Fig 8:**
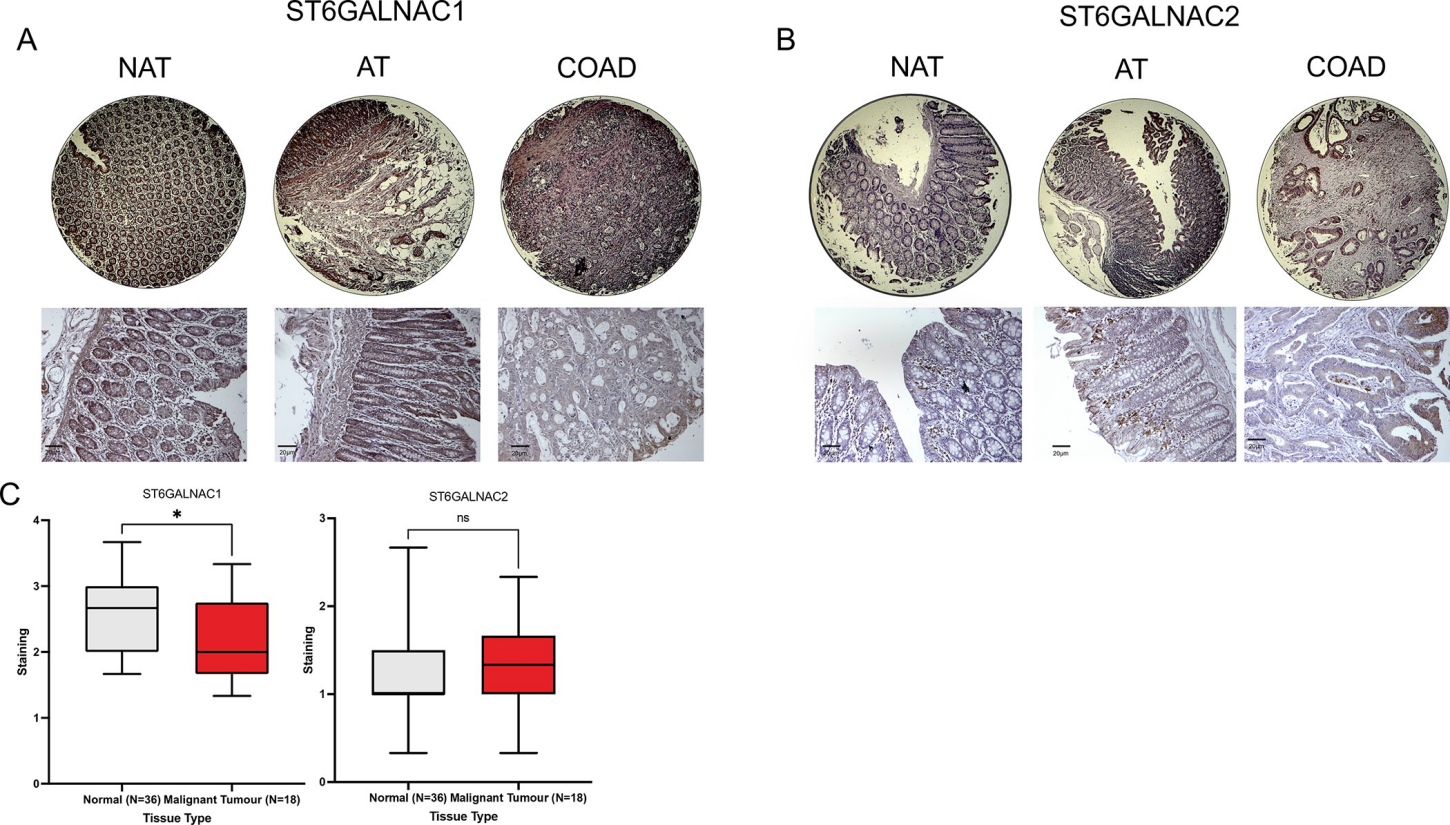
Low staining of *ST6GALNAC1* and *ST6GALNAC2* was observed in malignant tumour tissues. (**A**) IHC analysis was performed to investigate the presence of *ST6GALNAC1* in CRC tissues (BC05023a). Representative images of a total of 54 core tissue samples representing normal and malignant tissue cores were stained and visualised. (**B**) IHC analysis was performed to investigate the presence of *ST6GALNAC2* in CRC tissues (BC05023a). Representative images of a total of 54 core tissue samples, representing both normal and malignant tissue cores, were stained and visualised. (**C**) Quantitative analysis of the IHC staining for both *ST6GALNAC1* and *ST6GALNAC2* was performed for normal (clear) and malignant (red) tumour tissues. Unpaired student’s t-test. *P < 0.05.

IHC analysis was performed to investigate the presence of *ST6GALNAC1* and *ST6GALNAC2* proteins in CRC tissues (Fig 8). Minimal staining for *ST6GALNAC1* and *ST6GALNAC2* was observed in the malignant tumour tissue cores, with representative images of *ST6GALNAC1* and *ST6GALNAC2* staining in the tissue sections shown (Fig 8A and 8B). Quantitative analysis of the staining revealed a significant decrease in the expression of *ST6GALNAC1* between normal and malignant tumour cores, with non-significant staining found in both subgroups for *ST6GALNAC2* staining.

## Discussion

The current study has highlighted the multifaceted roles of both *ST6GALNAC1* and *ST6GALNAC2* and their collective association with the Siglec-15/Sia axis and its clinical relevance to CRC tumorigenesis. However, the regulatory mechanisms underlying their expression profiles remain unclear. Hence, we have identified possible regulatory mechanisms and miRNA candidates that seem to be involved in their regulation and, thereby, have some possible involvement in STn production.

The GeneMANIA database predicted *ST6GALNAC1* co-expression with *AGR2*. *AGR2* is localised to the endoplasmic reticulum (ER) and plays a crucial role in maintaining ER homeostasis via the formation, breakage, and isomerisation of the disulphide bonds involved in nascent protein maturation [27]. *AGR2* has also been found to be a prominent pro-tumorigenic gene that has consistently been associated with tumour onset and progression in a number of cancer types, including CRC [28,29]. Although AGR2 is predominantly localised in the ER, there is also evidence suggesting the extracellular secretion of AGR2 [30], where ER-resident AGR2 displays disparate O-glycosylation patterns compared to secreted AGR2 [31]. Similarly, *ST6GALNAC2* was shown to be co-expressed with and function alongside *AHSG*, an oncogene that is commonly associated with metabolic processes that have shown abnormal expression in multiple cancer types [32]. Furthermore, PPI interactions have highlighted that both sialyltransferases interact with a cluster of EEF1 proteins, particularly with EEF1A2. EEF1A2 serves an important role in modulating protein translation elongation and can play key roles in several biological processes [33]. To note, little is mentioned in the literature regarding the interactions between sialyltransferases and EEF1A2. However, *in vitro* studies have demonstrated that EEF1A2 is capable of regulating several signalling pathways, including PI3K/AKT and mTOR, via p53 inactivation [34]. Interaction with EEF1A2 could modulate the activity of ST6GALNAC1/ST6GALNAC2 and sialoglycan synthesis differently in normal and malignant tumour tissues.

Several previous studies have highlighted the aberrant expression of both sialyltransferase enzymes in multiple cancer types and the overexpression of the STn antigen [35,36]. However, TCGA genomic analysis highlighted significantly downregulated expression in the COAD subgroups for *ST6GALNAC1*. A previous study highlighted that increased *ST6GALNAC1* expression was only observed in the presence of M2 tumorigenic macrophages and CRC cells *in vitro*, which also stimulated the production of the STn antigen [37]. Moreover, another study outlined deficient mismatch repair (dMMR) CRC molecular subtype tumours displayed significantly downregulated expression of *GALNT6*, a prominent glycosyltransferase in glycan synthesis [38]. Consequently, this enhanced the pro-tumorigenic characteristics of SW480 cells *in vitro* and increased the expression of the Tn antigen, the precursor of the STn antigen. Additionally, GALNT6 was observed to interact with ST6GALNAC1 via our STRING analysis. This could suggest increased expression of the Tn antigen precursor molecule, and overexpression of STn may be independent of deregulated *ST6GALNAC1* expression in CRC and may require intercellular signalling to facilitate STn production in the TME. Further to this point, interactions with TAMs may be necessary for enhanced expression patterns that drive tumour heterogeneity within the TME and CRC progression. Further evidence also highlighted the downregulated expression of *ST6GALNAC2* in CRC cells [39]. This was also similarly observed in CRC tumour samples [40]. Possible epigenetic mechanisms and regulatory expression through miRNA activity may indicate dysregulated sialyltransferase activity. In addition, both Tn and STn antigens are more prevalent in CRC tumours, correlating with poor prognosis and reduced clinical survival outcomes in patients [40,41].

UALCAN datamining also indicated enhanced methylation of the promoter region of *ST6GALNAC2* in primary COAD tumours, suggesting a possible explanation for silencing gene expression. Additionally, enhanced CpG island methylation in the promoter regions of tumour-specific genes has also been associated with CRC tumorigenesis and further presents as one of the molecular subtypes of CRC [42]. Enhanced methylation and subsequent silencing of *ST6GALNAC2* expression could prevent its expression. Similarly, epigenetic modifications of histone proteins, including deacetylation may also repress *ST6GALNAC2* transcription [43].

Simultaneously, predicted miRNA candidates involved in sialyltransferase regulation have exhibited high binding affinities for the seed regions on the 3’ UTR of the mRNA target strand, suggesting a high likelihood of gene silencing [44]. Further to this point, full complementarity binding of the miRNA candidate to the target mRNA strand will lead to directed target degradation. In contrast, partial complementary binding can exhibit translational repression [45]. The role of pro-tumorigenic miR-21 has been characterised in the development and progression of multiple tumour malignancies, including CRC, which has also been highlighted as a potential tumour biomarker [46]. Furthermore, miR-21 was shown to induce advanced stage MSI type CRC tumours in conjunction with miR-335 activity, leading to poor prognosis in CRC patients [47]. In addition to this, miR-21 may play a role in tumour associated signalling pathways, including PI3K/AKT and TGF-β signalling [48,49]. This could suggest likely signalling pathways that stimulate CRC progression. Similarly, miR-30e has also been suggested as a potential biomarker for CRC development [50]. Moreover, miR-30e exhibited overexpression in CRC *in vitro* via stimulating the CXCL12 axis [51]. MiR-30e has also been demonstrated to be consistently deregulated in chemoresistant CRC patients [52]. The overexpression of miR-26b has also been observed in CRC and was identified to correlate with the expression of MMP-9 [53]. However, the role of miR-26b as an oncomir in CRC remains limited and requires further elucidation. Additionally, several miRNAs may facilitate the progression of the TME. Extracellular vesicles containing miR-21 stimulated tumour immune evasion in CRC via upregulated expression of the immune checkpoint PD-L1 in TAMs, thus enhancing tumour migration and invasion [54,55]. It has been understood that changes in miRNA expression patterns drive the onset of malignancies [56]. Moreover, the significance between these miRNA interactions on the seed region of the 3’ UTR and *ST6GALNAC1* expression may address its downregulated expression in CRC tumour malignancies. Additionally, it may suggest miRNAs are involved in modulating tumour heterogeneity in CRC and their activity enhanced pro-tumorigenic characteristics including treatment resistance, which inevitably results in poor survival outcomes. However, other epigenetic modifications as were observed with *ST6GALNAC2* may downplay the roles of these sialyltransferases in STn production. Therefore, this may provide insights into how *ST6GALNAC1* can become deregulated and may suggest the involvement of other sialyltransferase proteins to play a role in STn production.

We have correlated the role of *ST6GALNAC1* and *ST6GALNAC2* expression and the enrichment of specific gene sets associated with cancer hallmarks.

With respect to *ST6GALNAC1*, upregulation of EMT and MYC targets provide ample evidence, as reported in the literature, of its role as a possible oncogene, and may also outline possible signalling pathways through which to carry out tumorigenesis [57,58]. Of note, several downregulated mechanisms relating to *ST6GALNAC1* also involve IL-6-mediated JAK/STAT3 signalling. Pro-inflammatory stimulation of IL-6 underlines signalling via JAK/STAT3, which promote EMT in multiple cancer types [59]. JAK/STAT3 signalling pathways mediate the activation of EMT through a series of tyrosine and serine/threonine kinases [59]. A previous study associated EMT with CRC metastasis, emphasising crosstalk between CRC tumour cells and TAMs [60] *ST6GALNAC1* may facilitate TME heterogeneity following interactions with myeloid cells and EMT pathways. However, the observation of both this pathway and the inflammatory response showing downregulated enrichment supported our findings that EMT is mediated through another mechanism. This may suggest the activity of MYC targets in CRC progression. Disheveled-3 was shown to induce EMT in CRC progression mediated through MYC signalling and Wnt/β-catenin activity [61]. Moreover, syntrophin beta 1 (SNTB1) was shown to mediate EMT progression in CRC through a similar mechanism in knockdown studies [62]. Furthermore, enriched MYC targets may highlight the involvement of Zinc finger protein SNAI1 (SNAIL), a key regulator of the EMT process. Indeed, MYC was shown to induce SNAIL transcription and promote EMT via TGF-β action [63]. Characterisation of *ST6GALNAC1* and other gene targets in the MYC/Wnt/β-catenin crosstalk may propose a possible axis in CRC tumorigenesis.

*ST6GALNAC2* enrichment exhibited E2F and MYC targets. E2F target involvement in transcriptional regulation can be correlated with the development of multiple tumour malignancies [64]. Moreover, upregulation of E2F activity was exhibited upon the characterisation of deregulated CRC KRAS mutant tumours and CIN type tumours [65]. E2F expression is also directly associated with clinicopathological features of CRC and correlated with poor clinical outcomes [66]. In addition, synergistic signalling between E2F1/MYC can mediate epigenetic modulation in CRC with targeted inhibition of the axis inducing p-53 independent arrest [67]. Furthermore, a recent study identified the expression of E2F7 activating the transcription of enhancer of zeste homolog 2 (EZH2), thus inducing mTOR signalling in glioblastoma progression [68]. Upon identifying that mTOR signalling is also enriched with regard to *ST6GALNAC2*, this could possibly indicate that a similar interaction could induce the PI3K/AKT/mTOR signalling pathway, possibly through EEF1A2 and MYC. Similarly, another pro-tumorigenic signalling pathway associated with CRC progression had identified TGF-β signalling as significantly enriched. TGF-β signalling is associated with several characteristics of CRC tumours including EMT, angiogenesis and immunosuppression [69]. Interestingly, the downregulated enrichment of the inflammatory response and IFNγ response were revealed. The downregulation of both pathways may indicate CRC tumours associated with *ST6GALNAC2* expression is not dependent on inflammatory stimuli. Stimulated IFNγ response is an immunomodulatory mechanism directed against infection, inflammation and anti-tumour activity [70]. Further to this, several inflammatory mediated diseases, including inflammatory bowel disease and ulcerative colitis, could manifest to colitis-associated CRC as a consequence of chronic inflammation [71]. Therefore, the downregulated enrichment of both pathways in GSEA analysis could suggest *ST6GALNAC2* expression is not mediated by inflammatory stimuli and its expression profile may be context-dependent.

The role of sialyltransferases and their involvement with immune infiltration highlighted possible interactions within the TME. Although the exhibited SRCC scores outline a non-correlative relationship > 0.2, there is significance in the expression of *ST6GALNAC1* and activated CD4^+^ T-lymphocytes. A pan-cancer transcriptomics analysis identified monocytes and macrophages accounted for the largest proportion of tumour infiltrating myeloid cells [72]. The heterogeneity of the TME could possibly coincide with the pro-tumorigenic nature of several myeloid cell populations, including CD4^+^ T-lymphocytes [73]. A pan-cancer analysis of stromal heterogeneity was also able to predict the naïve CD4^+^ T-lymphocyte response to immunotherapeutic treatment [74]. Furthermore, monocyte depletion is also correlated to an immunosuppressive phenotype [75]. Moreover, a previous study highlighted the differential expression of *ST6GALNAC1* and its regulatory miRNAs, corresponding with intra-tumour heterogeneity in CRC metastasis [76]. Immunotherapeutic treatment has shown positive treatment responses in patients. However, further targeting additional myeloid cell populations may drive clinical effectiveness. Monocytes exhibit heterogeneity and plasticity within the TME through differentiation to the polarised M2 immunosuppressive phenotype. Blocking M2 polarisation or stimulating M1 monocyte differentiation may reduce the presence of TAMs, thus improving immunotherapeutic approaches in CRC [77]. Furthermore, stimulating CD4^+^ T-cells to Th1 cells could enhance CD8^+^ T-cell activation, promoting anti-tumour activity [78]. Although *ST6GALNAC1* shares a weak association with *TIGIT*, the sialyltransferase also shares significant expression with *TIGIT*, another immune checkpoint associated with cancer progression [76]. TIGIT has been shown to promote myeloid cell exhaustion, including CD8^+^ T-lymphocytes and enhanced expression profiles correlated with poor clinical outcomes in CRC [79]. This highlights the possibility of other immune checkpoint-related pathways contributing to tumour onset and tumour heterogeneity.

*ST6GALNAC2* had shown significant association with several subsets of myeloid cells and immune infiltration. By directly interacting with cellular components associated with the TME, their interaction could modulate the induction of metastasis and immune tolerance [80]. Additionally, solid tumours could display immunogenicity due to the heterogeneous nature of the TME, largely characterised by an immune-induced inflammatory phenotype [81]. The correlation between *ST6GALNAC2* expression and immune cell infiltration may offer insights for improving the efficacy of immunotherapeutic approaches. Further to this, reducing the sialylation of glycans will allow greater antigen recognition and promote anti-tumour immunity [82]. The binding of the STn antigen and Siglec-15 drives TGF-β secretion via monocytes and macrophages, possibly establishing tumoral recruitment and enhancing tumour heterogeneity via *ST6GALNAC2* overexpression [83]. Likewise, associations with immune checkpoints excluding Siglec-15 highlight potential combination treatment therapies for immune checkpoint blockade, although the literature associating *ST6GALNAC2* and immune checkpoints remains limited.

Several studies have indicated enhanced expression of *ST6GALNAC1* in several cancer tissues, including lung adenocarcinoma (LUAD), lung squamous cell carcinoma (LUSC), and clear-cell renal cell carcinoma (ccRCC) samples [84,85]. However, the outlined survival curves obtained via datamining demonstrated low *ST6GALNAC1* expression correlated with poor clinical outcomes across all survival criteria (p < 0.05). Similarly, this also corroborated the *in silico* data and IHC staining, outlining higher expression of *ST6GALNAC1* in normal tissues. This possibly highlights the involvement of other sialyltransferases in the production of the STn antigen, and inducing effective binding with Siglec-15.

Interestingly, high levels of *ST6GALNAC2* expression were statistically significant in all survival criteria (p < 0.05) and were associated with poor clinical outcomes. However, this was not seen at the gene and protein levels obtained via *in silico* datamining and IHC staining. One possible explanation could stem from silenced gene expression and modulated expression via miRNA binding. Previous studies have highlighted miR-182 and miR-135b were shown to modulate the expression of *ST6GALNAC2* via the PI3K/AKT pathway, promoting chemoresistance and tumour invasiveness [21,86]. This could prove similar as mTOR signalling was significantly enriched with regard to *ST6GALNAC2* expression.

To illustrate the role of the STn antigen in the CRC landscape, multiple studies have shown the abnormal expression of the STn antigen and its precursor Tn antigen molecule in CRC tumours [87]. Moreover, the expression of the STn antigen is not directly limited to solid CRC epithelial tumours. Circulating tumour cells of metastatic CRC patients expressed enhanced STn production, indicating its role in CRC metastasis to secondary organ sites [88]. Although the present study has identified possible miRNA candidates involved in the regulation of *ST6GALNAC1* and *ST6GALNAC2*, further sialyltransferase family members could play roles in CRC development. The STn antigen displays high binding affinity towards Siglec-15 [89]; however, other sialylated glycans have also displayed elevated binding affinity for Siglec-15 via *ST3GAL4* and *ST6GAL1* modulation [90]. Furthermore, GALNT enzymes highlighted by the STRING analysis are also heavily involved in GalNAc type-O glycosylation, this could suggest that members of the GALNT family including GALNT2 and GALNT6 facilitate glycan sialylation, including STn production. In addition, many GALNT genes have displayed dysregulated expression in CRC [91,92]. Therefore, this may suggest that downregulated *ST6GALNAC1* expression is independent to STn production. Elevated levels of STn and Tn antigens were also identified in CRC samples. However, their expression was induced by the loss of function of other glycosyltransferases, including C1GALT1 and COSMC, through promoter methylation or mutation [40]. Additionally, sulphation modifications of sialoglycans can also contribute to immune evasion and possible tumorigenesis. The overexpression of carbohydrate sulphotransferases, including *CHST1* and *CHST2*, can greatly induce the occurrence of hypersialylation and Siglec binding [93]. Furthermore, *CHST1* exhibited an accentuated effect on sialoglycan ligand binding and greatly impacted Siglec preference for O-glycans [94]. Further elucidation of the Siglec/Sia axis may highlight the role of other sialyltransferases in STn production.

Our findings highlighted a different view in the deregulated expression profiles of *ST6GALNAC1* and *ST6GALNAC2* in comparison to the literature. We believe that epigenetic modifications in conjunction with miRNA activity greatly impact gene expression and may outline sialyltransferase expression in relation to CRC as tumour specific. Furthermore, we provided insights into possible regulatory pathways and signalling pathways associated with their clinical relevance to CRC. Moreover, we believe that *ST6GALNAC1* and *ST6GALNAC2* is a minor player in the STn production in the case of CRC. The other sialyltransferase enzymes, such as *ST6GAL1*, *ST3GAL1*, and sulphotransferases *CHST1* might play a vital role in the production of the STn antigen in CRC.

Whilst a mutliomics approach identified the crosstalk of potential gene targets and regulatory pathways of sialyltransferase expression from the integration of several key datasets. There are significant limitations to consider when utilising multiple databases. Firstly, the well-defined regulatory pathways identified through *in silico* analysis may not fully highlight the interplay of sialylation and sialyltransferase activity without experimental validation to directly support their potential impact on cellular behaviour [95,96]. Further to this point, variation in methodologies may introduce false discoveries which highlight difficulties for data comparability [97]. Moreover, limited sample size and availability may impact *in silico* software thus creating challenges that require further sophisticated data mining tools. Additionally, there are also difficulties in histological staining for truncated O-glycan structures. Antibodies are required to have high specificity for antigen staining to prevent non-specific cross-reactive staining of similarly structured glycans [98]. However, addressing these limiting factors in association with experimental validation with further studies will provide more robust data interpretation and elucidation of sialyltransferase regulation.

In conclusion, the present study has predicted possible oncomirs involved in the regulation of *ST6GALNAC1* with high binding affinities, all of which displayed significantly upregulated expression in CRC tumours. However, downregulated expression of *ST6GALNAC1* in CRC might highlight the involvement of other sialyltransferases in the production of the STn antigen and have suggested the interplay of several key sialyltransferases that could play a role. Moreover, we have identified several regulatory signalling pathways that have highlighted the clinical relevance of both *ST6GALNAC1* and *ST6GALNAC2* to CRC progression. Further elucidation of this pathway will give significant insights into the regulation of the Siglec-15/Sia axis.

## Supporting information

S1 Table Sialyltransferase predicted PPI relationships and targets. STRING network analysis for predicted protein-protein interactions and direct associations obtained via computational data mining for ST6GALNAC1 and ST6GALNAC2 sialyltransferase enzymes. The obtained framework was developed with a high confidence interval ≥0.700.
(DOCX)

S2 Table Predicted miRNA binding targets for sialyltransferase gene regulation. *In silico* analysis to determine common miRNA targets predicted to modulate *ST6GALNAC1* via Venn diagrams.
(DOCX)

S3 Table Enriched signalling pathways corresponding with cancer hallmarks relating to *ST6GALNAC1* expression. GSEA enrichment scores for the association of ST6GALNAC1 with cancer hallmarks *(*FDR < 0.25 and p < 0.05*)*.
(DOCX)

S4 Table Enriched signalling pathways corresponding with cancer hallmarks relating to *ST6GALNAC2* expression. GSEA enrichment scores for the association of ST6GALNAC2 with cancer hallmarks *(*FDR < 0.25 and p < 0.05*)*.
(DOCX)

S1 Fig *AHSG* exhibits upregulated expression in CRC tumours. UALCAN genomic data was used to determine the gene expression of STRING protein targets *AGR2* (ENSG00000106541) and *AHSG* (ENSG00000145192) and were compared between normal and tumour cohorts.
(TIF)

S2 Fig The promoter region for ST6GALNAC2 is highly methylated in COAD tumours. UALCAN TCGA genomic data was used to identify promoter methylation of *ST6GALNAC1* (ENSG00000070526) and *ST6GALNAC2* (ENSG00000070731) and were compared between normal and colon adenocarcinoma (COAD) cohorts.
(TIF)

S3 Fig The association between ST6GALNAC1 and ST6GALNAC2 gene expression was considered significant. The Pearson correlation coefficient comparing *ST6GALNAC1* and *ST6GALNAC2* gene expression was determined.
(TIF)

S4 Fig Common miRNA targets between predicted PPI targets associated with ST6GALNAC1 and ST6GALNAC2. (**A**) No common miRNAs were predicted between *AGR2*, *AHSG*, *ST6GALNAC1* and *ST6GALNAC2*. MiR-432 was common between AGR2, ST6GALNAC1 and ST6GALNAC2. (**B**) Common miRNAs were determined between *GALNT3*, *GALNT8*, *B3GNT6* and *ST6GALNAC1*. MiR-30a-5p was predicted as the common miRNA between all four genes. (**C**) Common miRNAs were determined between *AHSG*, *C1GALT1C1*, *C1GALT1* and *ST6GALNAC2*. No common miRNAs were determined between each gene.
(TIF)

## Abbreviations

AGR2: anterior gradient 2
AHSG: α-2-HS-glycoprotein
ALCC: average local clustering coefficient
AT: cancer adjacent colon tissue
C1GALT1: Core-1 synthase-glycoprotein-*N*-acetylgalactosamine 3-b-galactosyltransferase- 1
C1GALT1C1: C1GALT1 Specific Chaperone 1
ccRCC: clear-cell renal cell carcinoma
CI: confidence interval
CIMP: CpG island methylation
CIN: chromosomal instability
COAD: colon adenocarcinoma
CRC: colorectal cancer
CTLA4: Cytotoxic T-lymphocyte associated protein 4
EEF: eukaryotic elongation factor
EEF1A2: Elongation factor 1-a 2
EMT: epithelial-mesenchymal transition
ER: endoplasmic reticulum
EZH2: Enhancer of zeste homolog 2
FDR: false discovery rate
FTC: follicular thyroid carcinomas
GALNT: *N*-acetylgalactosaminyltransferase
GALNT: N-acetylgalactosaminyltransferase
GSEA: Gene Set Enrichment Analysis
HR: hazard ratio
IHC: immunohistochemistry
LAG3: Lymphocyte-activation gene 3
LUAD: lung adenocarcinoma
LUSC: lung squamous cell carcinoma
miRNAs: microRNAs
MSI: microsatellite instability
NAT: adjacent normal colon tissue
OS: overall survival
PD-L1: programmed death-ligand 1
PPI: protein-protein interactions
PPS: post-progression survival
RECIST: response evaluation criteria in solid tumours
RFS: relapse-free survival
RT: room temperature
Sfold: statistical folding of nucleic acids and studies of regulatory RNAs
Siglec: Sialic acid-binding immunoglobulin-type lectin
SNAIL: Zinc finger protein SNAI1SNTB1—syntrophin beta 1
SRCC: spearman’s rank correlation coefficient
STn: Sialyl Tn
STRING: Search Tool for the Retrieval of Interacting Genes/Proteins
TAMs: tumour-associated macrophages
TIGIT: T-cell immunoreceptor with Ig and ITIM domains
TILs: tumour-infiltrating lymphocytes
TIMER: tumour IMmune Estimation Resource analysis
TME: tumour microenvironment
TPM: transcript per million
Tregs: regulatory T-cells
UTR: untranslated region
