# Supplementary material for: Differential expression of ST6GALNAC1 and ST6GALNAC2 and their clinical relevance to colorectal cancer progression
Source: PLoS One. 2024 Sep 30;19(9):e0311212. doi: 10.1371/journal.pone.0311212 (PMC11441655; doi:10.1371/journal.pone.0311212)
Supplement: S1 Table — STRING network analysis for predicted protein-protein interactions and direct associations obtained via computational data mining for ST6GALNAC1 and ST6GALNAC2 sialyltransferase enzymes. The obtained framework was developed with a high confidence interval ≥0.700. (DOCX) [file pone.0311212.s001.docx]

|  | Number of nodes | Number of edges | Average node degree | Average local clustering coefficient | Expected number of edges | PPI enrichment p-value |  |
| --- | --- | --- | --- | --- | --- | --- | --- |
| ST6GALNAC1 | 31 | 127 | 8.19 | 0.83 | 31 | <1.0E-16 |  |
|  |  |  |  |  |  |  |  |
|  |  |  |  |  |  |  |  |
|  |  |  |  |  |  |  |  |
|  |  |  |  |  |  |  |  |
| ST6GALNAC2 | 26 | 64 | 4.92 | 0.708 | 36 | 1.09E-05 |  |
