# Supplementary material for: Differential expression of ST6GALNAC1 and ST6GALNAC2 and their clinical relevance to colorectal cancer progression
Source: PLoS One. 2024 Sep 30;19(9):e0311212. doi: 10.1371/journal.pone.0311212 (PMC11441655; doi:10.1371/journal.pone.0311212)
Supplement: S2 Table — In silico analysis to determine common miRNA targets predicted to modulate ST6GALNAC1 via Venn diagrams. (DOCX) [file pone.0311212.s002.docx]

|  | Predicted candidate miRNAs |  |
| --- | --- | --- |
| ST6GALNAC1 | hsa-miR-204-5p, hsa-miR-211-5p, hsa-miR-26a-5p, hsa-miR-30a-5p, hsa-miR-361-5p, hsa-miR-432-5p  hsa-miR-526b-5p, hsa-miR-587, hsa-miR-605-5p, hsa-miR-623, hsa-miR-922 |  |
|  |  |  |
|  |  |  |
|  |  |  |
