# Supplementary material for: Differential expression of ST6GALNAC1 and ST6GALNAC2 and their clinical relevance to colorectal cancer progression
Source: PLoS One. 2024 Sep 30;19(9):e0311212. doi: 10.1371/journal.pone.0311212 (PMC11441655; doi:10.1371/journal.pone.0311212)
Supplement: S3 Table — GSEA enrichment scores for the association of ST6GALNAC1 with cancer hallmarks (FDR < 0.25 and p < 0.05). (DOCX) [file pone.0311212.s003.docx]

|  | Enriched Pathway | Effect on gene regulation | Normalised Enrichment Score (NES) | FDR q-value |
| --- | --- | --- | --- | --- |
| ST6GALNAC1 | Epithelial-Mesenchymal transition | Upregulated | 2.1449792 | 0.0024 |
|  | Myogenesis | Upregulated | 1.7376845 | 0.024640111 |
|  | MYC targets V1 | Upregulated | 1.7886475 | 0.025864933 |
|  | Pancreas - β cells | Downregulated | -2.005207 | 0.0034156502 |
|  | Inflammatory response | Downregulated | -1.7572728 | 0.07413546 |
|  | Fatty acid metabolism | Downregulated | -1.6966281 | 0.08710867 |
|  | Late Oestrogen response | Downregulated | -1.6314135 | 0.122363426 |
|  | KRAS signalling - downregulation | Downregulated | -1.6111323 | 0.11502332 |
|  | IL6/JAK/STAT3 signalling | Downregulated | -1.5580528 | 0.15035994 |
|  | KRAS signalling - upregulation | Downregulated | -1.492585 | 0.21481581 |
|  | HEME metabolism | Downregulated | -1.4515557 | 0.24811935 |
