# Supplementary material for: Differential expression of ST6GALNAC1 and ST6GALNAC2 and their clinical relevance to colorectal cancer progression
Source: PLoS One. 2024 Sep 30;19(9):e0311212. doi: 10.1371/journal.pone.0311212 (PMC11441655; doi:10.1371/journal.pone.0311212)
Supplement: S4 Table — GSEA enrichment scores for the association of ST6GALNAC2 with cancer hallmarks (FDR < 0.25 and p < 0.05). (DOCX) [file pone.0311212.s004.docx]

|  | Enriched Pathway | Effect on gene regulation | Normalised Enrichment Score (NES) | FDR q-value |
| --- | --- | --- | --- | --- |
| ST6GALNAC2 | E2F Targets | Upregulated | 5.906333 | 0.0 |
|  | MYC targets V1 | Upregulated | 4.5617013 | 0.0 |
|  | MYC targets V2 | Upregulated | 3.5422199 | 0.0 |
|  | G2M checkpoint | Upregulated | 2.9441366 | 0.0 |
|  | Peroxisome | Upregulated | 2.5570476 | 0.0 |
|  | MTORC1 Signalling | Upregulated | 2.4542193 | 0.0 |
|  | Bile Acid metabolism | Upregulated | 1.9872706 | 0.0029597476 |
|  | Mitotic signalling | Upregulated | 1.6982983 | 0.024412982 |
|  | TGF-β Signalling | Upregulated | 1.6413039 | 0.02922038 |
|  | Protein secretion | Upregulated | 1.4845706 | 0.04508288 |
|  | KRAS signalling - Up | Downregulated | -1.6662811 | 0.16640176 |
|  | Allograft Rejection | Downregulated | -1.6352895 | 0.109008245 |
|  | Epithelial to Mesenchymal Transition | Downregulated | -1.6047972 | 0.10321164 |
|  | Inflammatory response | Downregulated | -1.5750812 | 0.102057174 |
|  | KRAS signalling -DN | Downregulated | -1.4115723 | 0.30620778 |
|  | Interferon gamma response | Downregulated | -1.3972876 | 0.28351775 |
|  | Late Oestrogen response | Downregulated | -1.3743881 | 0.28962904 |
