# Supplementary figures and images for: Differential expression of ST6GALNAC1 and ST6GALNAC2 and their clinical relevance to colorectal cancer progression
Source: PLoS One. 2024 Sep 30;19(9):e0311212. doi: 10.1371/journal.pone.0311212 (PMC11441655; doi:10.1371/journal.pone.0311212)

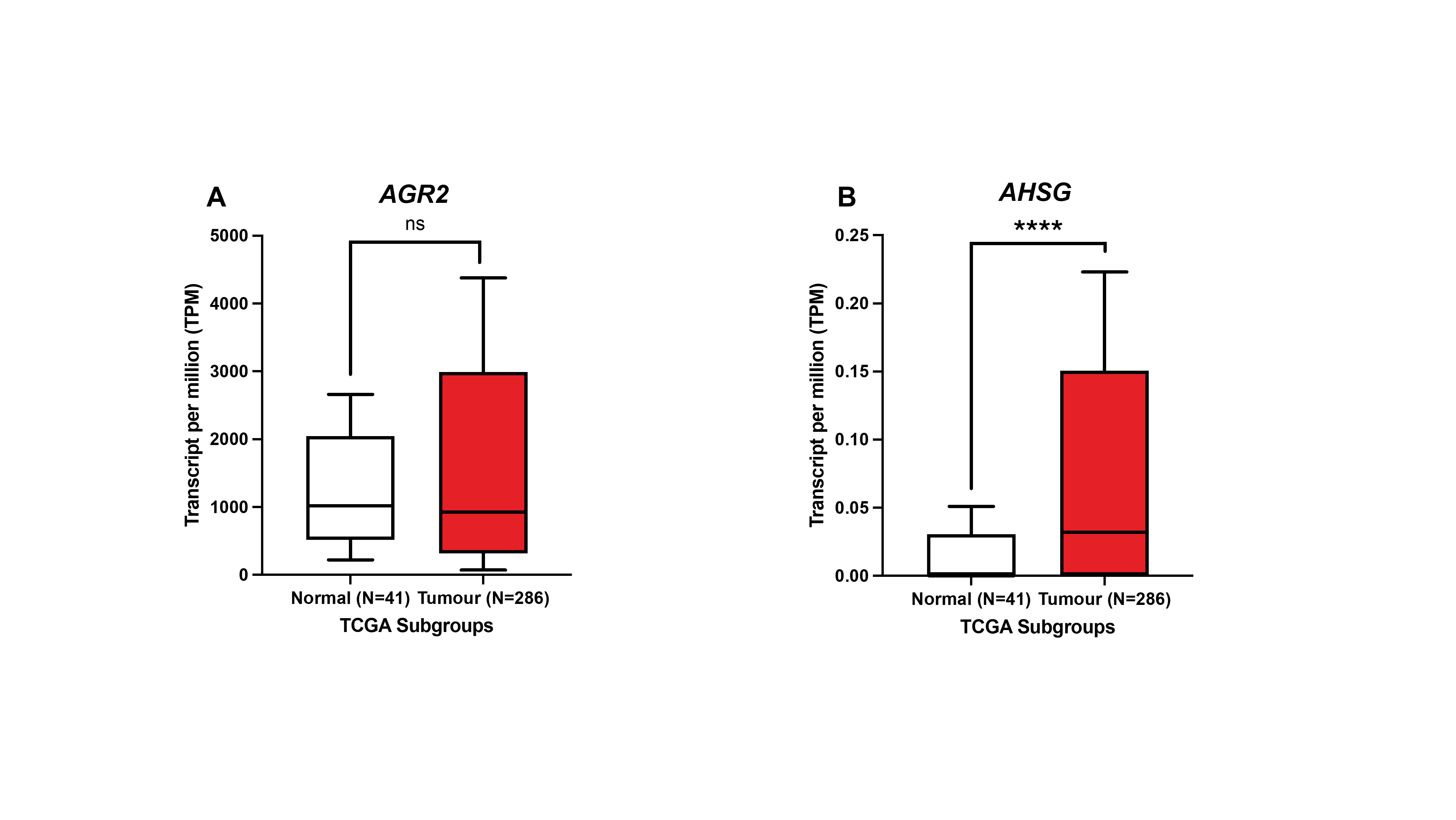

Supplement: S1 Fig — UALCAN genomic data was used to determine the gene expression of STRING protein targets AGR2 (ENSG00000106541) and AHSG (ENSG00000145192) and were compared between normal and tumour cohorts. (TIF) [file pone.0311212.s005.tif]

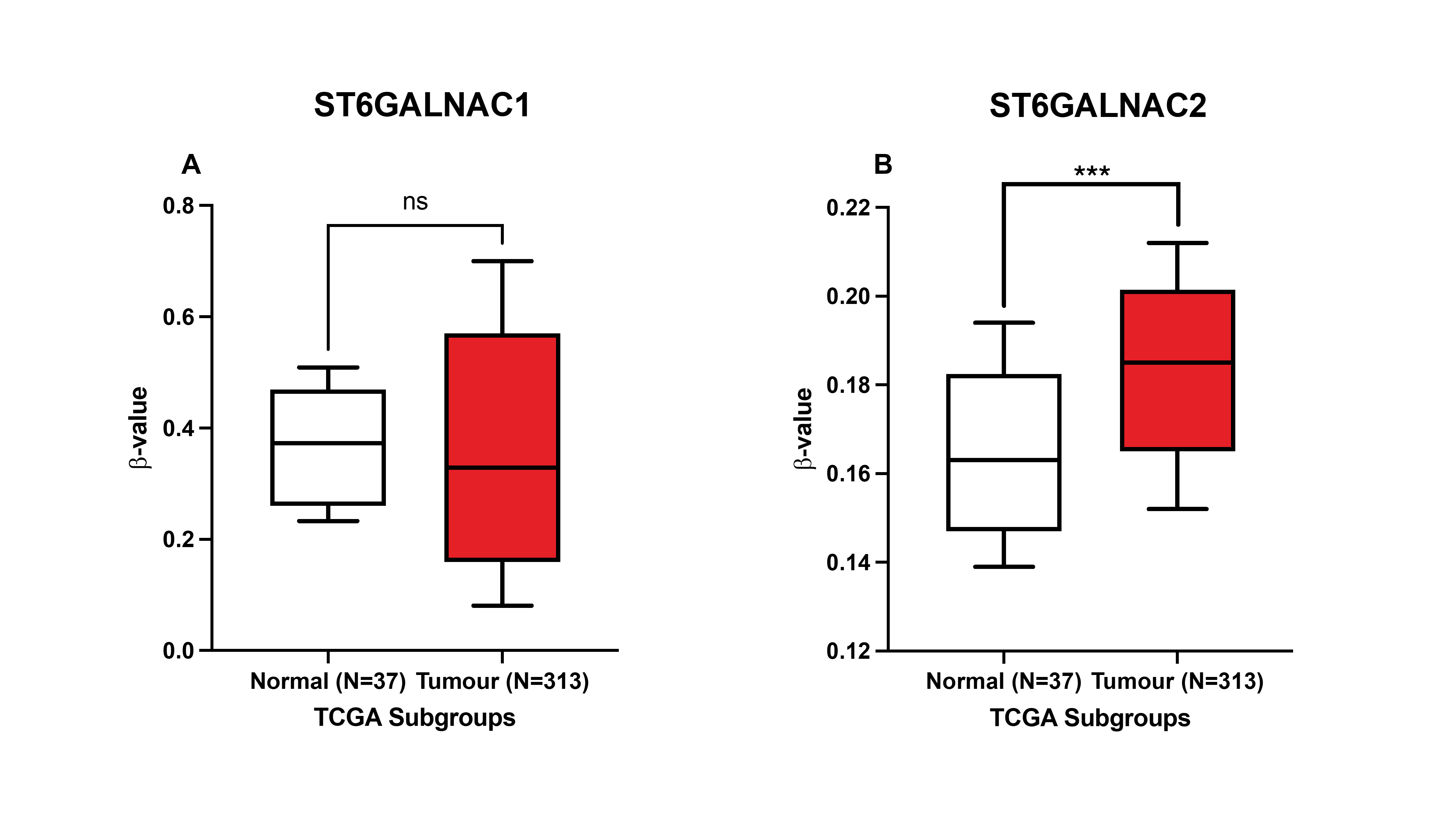

Supplement: S2 Fig — UALCAN TCGA genomic data was used to identify promoter methylation of ST6GALNAC1 (ENSG00000070526) and ST6GALNAC2 (ENSG00000070731) and were compared between normal and colon adenocarcinoma (COAD) cohorts. (TIF) [file pone.0311212.s006.tif]

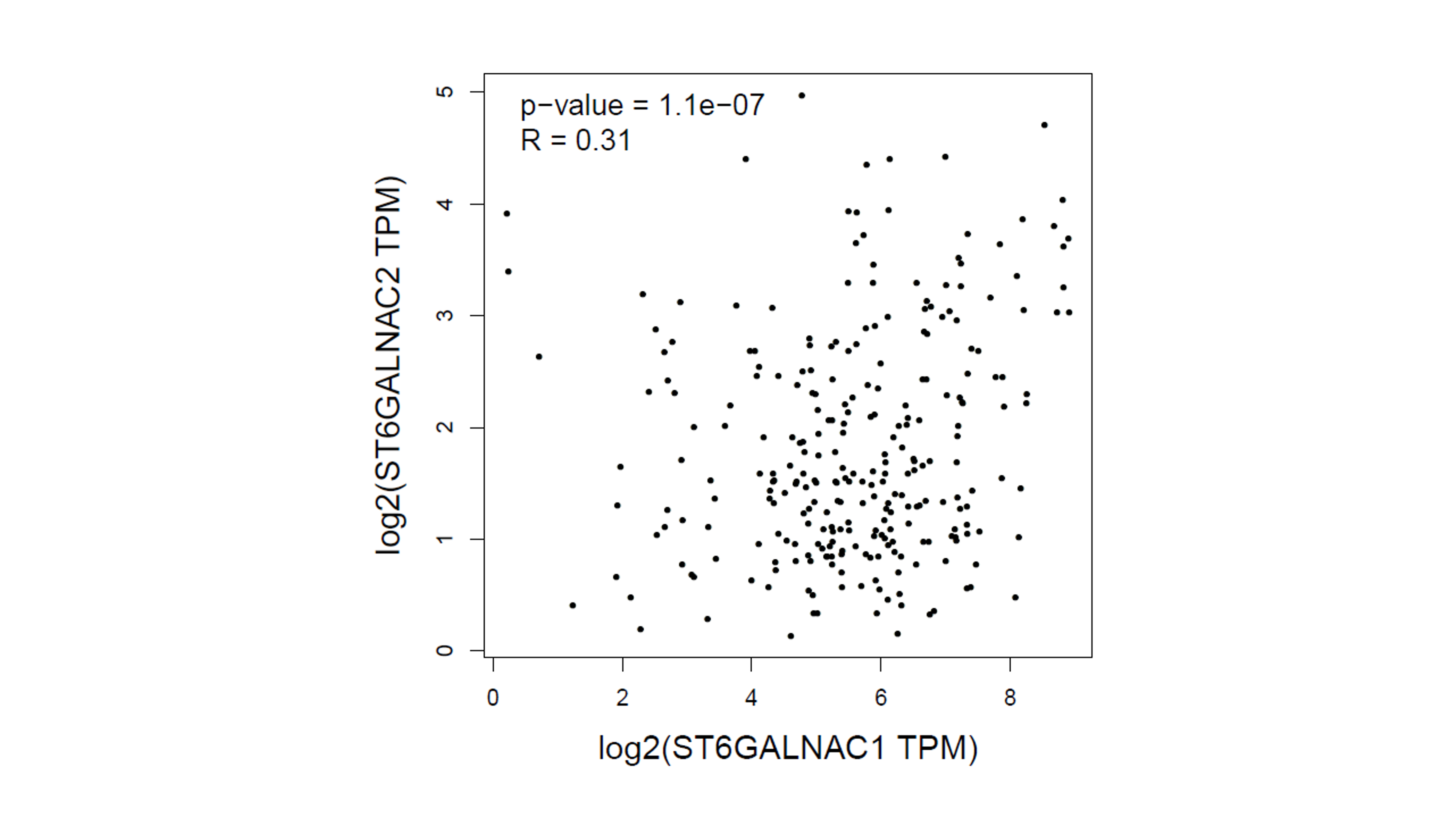

Supplement: S3 Fig — The Pearson correlation coefficient comparing ST6GALNAC1 and ST6GALNAC2 gene expression was determined. (TIF) [file pone.0311212.s007.tif]

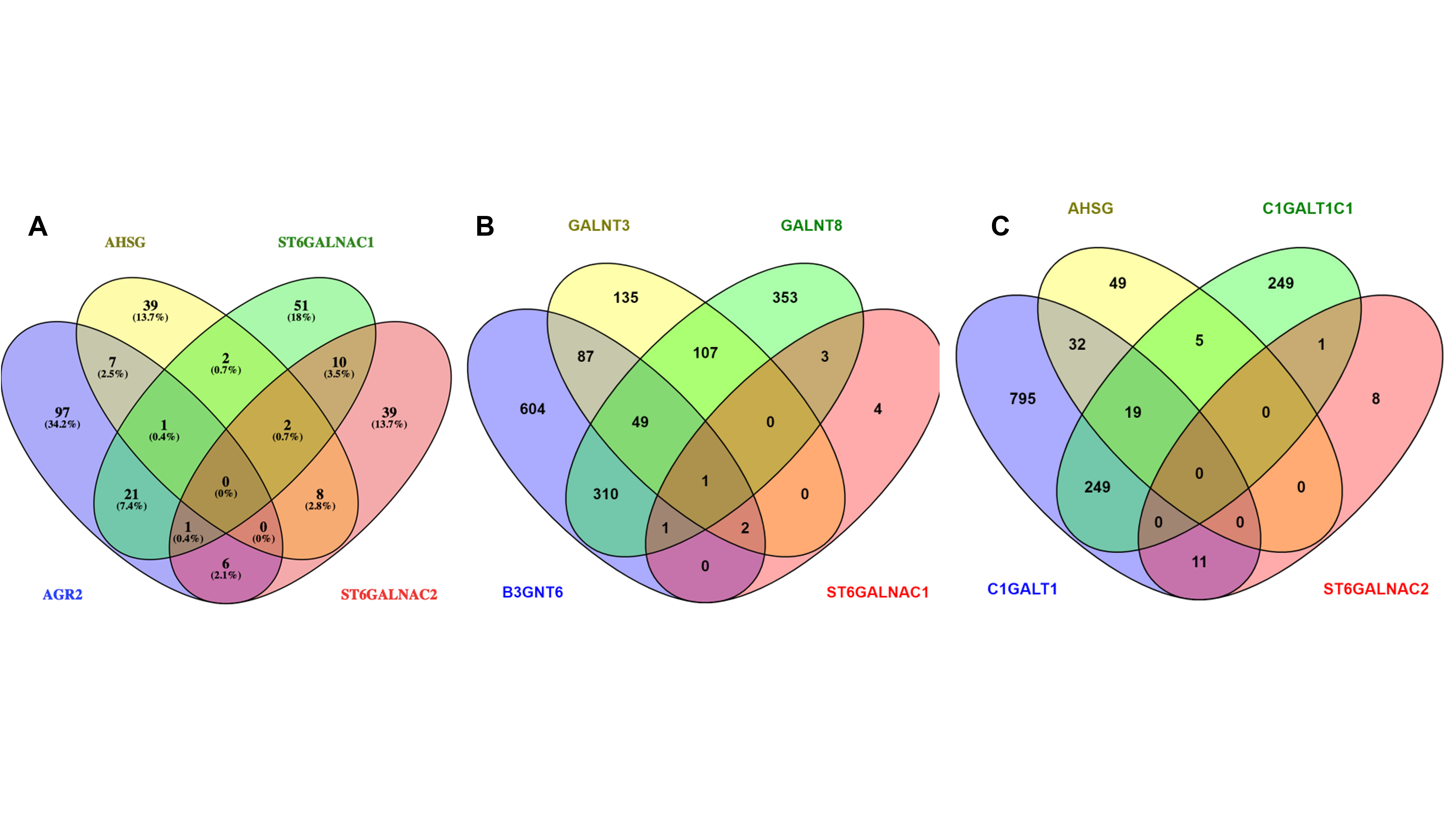

Supplement: S4 Fig — (A) No common miRNAs were predicted between AGR2, AHSG, ST6GALNAC1 and ST6GALNAC2. MiR-432 was common between AGR2, ST6GALNAC1 and ST6GALNAC2. (B) Common miRNAs were determined between GALNT3, GALNT8, B3GNT6 and ST6GALNAC1. MiR-30a-5p was predicted as the common miRNA between all four genes. (C) Common miRNAs were determined between AHSG, C1GALT1C1, C1GALT1 and ST6GALNAC2. No common miRNAs were determined between each gene. (TIF) [file pone.0311212.s008.tif]
